# A Novel Fiber Optic Surface Plasmon Resonance Biosensors with Special Boronic Acid Derivative to Detect Glycoprotein

**DOI:** 10.3390/s17102259

**Published:** 2017-10-01

**Authors:** Yang Zhang, Fang Wang, Siyu Qian, Zexu Liu, Qiao Wang, Yiying Gu, Zhenlin Wu, Zhenguo Jing, Changsen Sun, Wei Peng

**Affiliations:** School of Physics and Optoelectronic Technology, Dalian University of Technology, 2 Linggong Road, Ganjingzi District, Dalian 116024, China; yangzhang@dlut.edu.cn (Y.Z.); w15122514950@163.com (F.W.); qiansy321@163.com (S.Q.); liuzexu@mail.dlut.edu.cn (Z.L.); wangqiao@dlut.edu.cn (Q.W.); yiyinggu@dlut.edu.cn (Y.G.); zhenlinwu@dlut.edu.cn (Z.W.); jingzg@dlut.edu.cn (Z.J.); suncs@dlut.edu.cn (C.S.)

**Keywords:** tilted fiber Bragg grating, surface plasmon resonance, boronic acid, glycoprotein

## Abstract

We proposed and demonstrated a novel tilted fiber Bragg grating (TFBG)-based surface plasmon resonance (SPR) label-free biosensor via a special boronic acid derivative to detect glycoprotein with high sensitivity and selectivity. TFBG, as an effective sensing element for optical sensing in near-infrared wavelengths, possess the unique capability of easily exciting the SPR effect on fiber surface which coated with a nano-scale metal layer. SPR properties can be accurately detected by measuring the variation of transmitted spectra at optical communication wavelengths. In our experiment, a 10° TFBG coated with a 50 nm gold film was manufactured to stimulate SPR on a sensor surface. To detect glycoprotein selectively, the sensor was immobilized using designed phenylboronic acid as the recognition molecule, which can covalently bond with 1,2- or 1,3-diols to form five- or six-membered cyclic complexes for attaching diol-containing biomolecules and proteins. The phenylboronic acid was synthetized with long alkyl groups offering more flexible space, which was able to improve the capability of binding glycoprotein. The proposed TFBG-SPR sensors exhibit good selectivity and repeatability with a protein concentration sensitivity up to 2.867 dB/ (mg/mL) and a limit of detection (LOD) of 15.56 nM.

## 1. Introduction

Optical fiber sensing technology, combined with the surface plasmon resonance (SPR) effect, enhances the measurement sensitivity of a surrounding refractive index (SRI) and enables the label-free detection of binding interactions on fiber surfaces [1,2,3,4]. Optical fiber SPR sensors possess advantages of both fiber sensors and traditional SPR sensors. Compared with traditional prism configuration, fiber optical SRP sensors are more flexible, are compact, and are can even be inserted into human tissue or blood vessels for real-time analysis or monitoring. It has been demonstrated in recent years that tilted fiber Bragg grating (TFBG)-based SPR sensors offer a novel and unique platform for simple, low-cost, and highly sensitive biomedical and chemical detection [5,6,7]. In a tilted fiber Bragg grating (TFBG), a number of cladding modes, whose optical field extends beyond the fiber to interact with the fiber’s surroundings, are able to excite the SPR under certain conditions; thus, the surrounding RI can be accurately detected by measuring the variation of transmitted spectra of the TFBG. TFBG-SPR sensors, compared with the geometry-modified or uncommon fiber-based optic SPR sensors operating within the visible wavelength region, typically operate at a 1550 nm wavelength (the communication band), which not only shows a high figure of merit (FOM) due to the narrow resonance bandwidth, but also enables a relatively low-cost and mature fiber sensor interrogation system. Moreover, unlike other fiber optic structures such as etched fiber Bragg grating (FBG), tapered fiber, side-polished fiber, or D-shaped fiber-based grating, TFBG-based SPR sensors can be manufactured with low cost using a common single mode fiber without compromising the mechanical and geometric integrity of the fiber [8,9]. A stable SPR wave can be easily excited by a nano-scale metal film (usually Au or Ag) coated on the TFBG surface. After the chemical decoration of the recognition molecule, the sensor can selectively detect analytes that perturb the local refractive index of the dielectric and plasmon phase velocity.

Glycoprotein is a vital physiological active object, which is made from many covalent forms of polypeptide and oligosaccharide. In recent years, it has been demonstrated that glycoprotein is related to many diseases, such as diabetes, neurodegenerative disorders, cancer, and metastasis [10,11,12]. The glycoprotein of the cell surface can fall into blood circulation, so the detection of glycoprotein has been a powerful tool for disease diagnosis. Traditional techniques for glycoprotein detection include fluorescent probes, glucose oxidase sensors, and lectin-aggregated colloides [13,14,15,16]. However, most of these methods are vulnerable to sample contamination. Therefore, the development of systems with a low cost, a compact size, and good stability for highly sensitive and selective detection of glycoprotein is important for monitoring the saccharides in cell-to-cell interactions and biological recognition. 

In this paper, to detect glycoprotein with high sensitivity and selectivity, we present a TFBG-SPR sensor loaded with a phenylboronic acid derivative (PBA) as a receptor molecule on the sensing region. Boronic acid can covalently bond with 1,2- or 1,3-diols configuration (e.g., glycoprotein) by forming five- or six-membered cyclic complexes [17]. It has been used for various applications, such as the recognition matrix for chemo/biosensing of diol-containing biomolecules [18,19,20,21]. Instead of using commercial available short-chain 4-mercaptophenylboronic acid, we synthesized a phenylboronic acid derivative with a long chain that offers more binding sites to improve the binding capability of the glycoprotein [22]. In the experiments, different proteins, including glycoproteins and non-glycoprotein, were measured to verify the selectivity of the sensor, and the regeneration capacity was also investigated. Moreover, different glycoproteins with different molecular weights were compared to investigate that the sensor had different responses to the volume of the glycoprotein. Finally, the proposed TFBG-SPR sensor achieved good repeatability, protein concentration sensitivity, and detection limitation.

## 2. Experimental System and Methods

**Figure 1:**
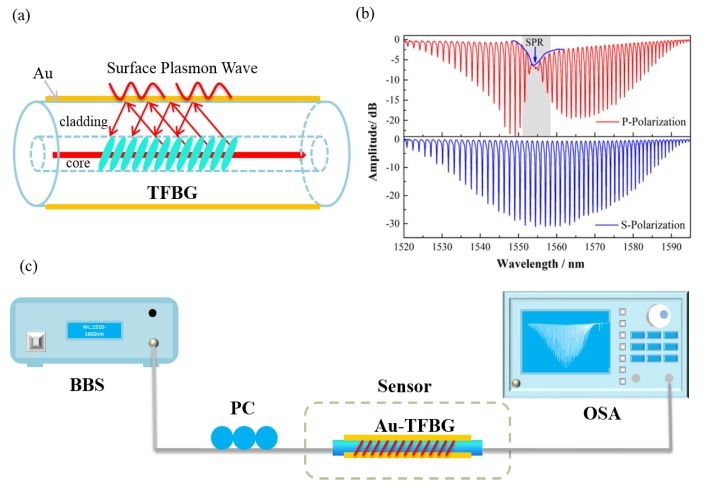
(**a**) Schematic diagram of the tilted fiber Bragg grating (TFBG)-based surface plasmon resonance (SPR) (TFBG-SPR) sensor. (**b**) Transmission spectra of the sensor under P and S polarization; SPR occurs when P-polarized light strikes an electrically conducting gold layer at the interface. (**c**) Experimental setup for detection; the TFBG was excited by a broadband source (BBS), and its transmission spectrum was monitored by an optical spectrum analyzer (OSA).

The schematic configuration of the proposed sensor is shown in Figure 1a. TFBGs are a special kind of fiber Bragg gratings, in which the index variation in the fiber core is at an angle to the fiber axis. The function of the angle is to couple the light from the core mode to higher-order cladding modes and to break the cylindrical symmetry of the coupling process relative to the state of the guided light polarization. Consequently, TFBGs can easily excite the higher-order cladding modes, enhancing the interaction between the light and the fiber surface surrounds. SPR occurs when P-polarized light, under conditions of total internal reflection (TIR), impinges onto an electrically conducting gold layer at the interface between two media of opposite dielectric constants, e.g., the water or solution with a positive dielectric constant and a metal film with a negative dielectric constant. When complex biochemical reactions occur on the surface of the sensor, the interaction, mostly the changes in refractive index, can be detected accurately by measuring the wavelength and amplitude variation of certain high-order cladding modes next to the SPR region [23,24,25,26,27]. Figure 1b presents the transmission spectrum of a TFBG when coated with a 50 nm thick Au film under P and S polarization. In the experiments, TFBGs were inscribed in a standard single mode fiber (SMF-28 Corning telecom fiber, with high-pressure hydrogen loading for two weeks) using a 248 nm UV excimer laser (BraggStar200, Tuilaser Corp., Munich, Germany ) and the phase-mask method. During the fabrication, the laser pulse was set to 6 mJ/150 Hz and 15 mm long TFBG was made by the scanning technique. The polarization of the injected light was controlled by adjusting the three modules of a polarization controller (PC), PR2000 from JDSU, which consisted of a polarizer, a half-wave plate, and a quarter-wave plate. Any polarization state could be generated by rotating the polarizer and the two plates. The gold film thickness was selected based on the thickness dependence of the TFBG-SPR response investigated in [8]. We deposited a gold film around the fiber surface by using an ion sputtering instrument K575X from Emitech Corp. For the 125 µm thick fiber that we used in the experiments, there was no gold film deterioration observed in the experiments. Figure 1b clearly shows the TFBG spectrum under two orthogonal polarizations and P-polarized light stimulated the SPR effect on the sensor. The experimental system for detecting the glycoprotein is shown in Figure 1c. The sensor was illuminated by a broadband source (BBS, A0002 from HaoYuan Photoelectric Corp., Shenzhen, China) in the C + L band with 1510–1610 nm wavelength range, and the transmission spectrum was monitored by an optical spectrum analyzer (OSA, AQ6370 from Tokogawa, Tokyo, Japan) with a wavelength resolution of 0.02 nm and an amplitude resolution of 0.02 dB.

**Figure 2:**
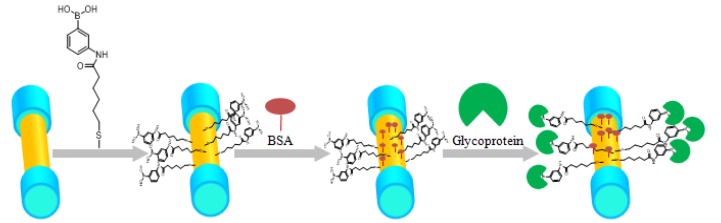
Decoration procedure on the SPR sensing region and application in glycoprotein detection, the sensor was dipped in boronic acid derivative for 24 h, which the TFBG-SPR would self-assemble with the molecules and then block the unreacted carboxyl by dipping the sensor to the BSA, different concentrations of glycoprotein, and different kinds were detected in the end.

The whole protein specific identification procedure and the immobilization process for boronic acid derivative and glycoprotein were performed as depicted in Figure 2. The bovine serum albumin (BSA), glycoprotein (Con A, Rnase B), and Rnase A were obtained from Aladdin (Shanghai, China). PBS buffer (10 mM HEPES, 150 mM NaCl, 1 mM CaCl_2_, 1 mM MnCl_2_, adjusted to pH 7.4) was used to establish a reference baseline. First, to clean the surface of the sensor, the Au-coated SPR-TFBG probe was rinsed with ethanol and deionized water. Then, the sensor was dipped in boronic acid derivative, which was dissolved in the ethanol for 24 h so that the molecules were self-assembled onto the SPR-TFBG. After these pretreatments, the sensing element was fixed in a micro-fluidic channel that provided precise control over the flow rate. Reagents (1 mg/mL BSA, 1 mg/mL RNase B, 1 mg/mL RNase A, and 1 mg/mL Con A) were dissolved in the PBS buffer. In this experiment, the flow rate produced by a peristaltic pump was set to 50 μL/min. The PBS buffer was firstly pumped into the flow cell to obtain a stable baseline. After that, BSA (bovine serum albumin, 1 mg/mL) flowed over the surface to block the unbounded binging sites on the sensor surface. We monitored the specific interaction between boronic acid derivative and glycoprotein after the functionalization of the sensing surface. 

The recognition molecule used in the experiments was (3-((11-mercaptoun-decanamido) methyl) phenyl) boronic acid (PBA with long chain), which is different from common short chain PBA such as 4-mercaptophenylboronic [22]. In principle, the long-chain PBA offers more binding space for glycoprotein than the short-chain PBA. The long-chain PBA offers a greater probability and capability of binding positions in the conformation of proteins that cannot be reached by the short-chain PBA. Additionally, when PBA is directly attached to the sensor surface, a long chain and flexible spacer will separate PBA from the surface, and the protein will then have more freedom to bind with PBA. To verify the selectivity of boronic acid derivative, we first detected different proteins (glycoprotein and non-glycoprotein) simultaneously. Then, the analyte (Con A) was diluted to different concentrations in the PBS buffer to measure the sensitivity and the limit of detection (LOD) of the sensor. Finally, a high-refractive-index solution (ethanol: PBS = 3:1, pH = 3) was used as a regeneration solution to remove the analyte from the sensor surface. During our experiment, the wavelength and amplitude of the cladding modes near the SPR absorption were monitored in real time.

## 3. Results and Discussion

**Figure 3:**
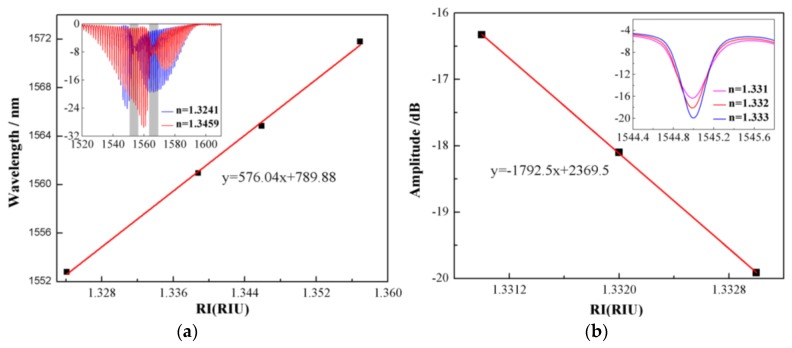
(**a**) The wavelength response of TFBG-SPR, attained by extracting the wavelength shifts of the most attenuated resonance in the spectrum (i.e., the SPR resonance). The measured sensitivity is 576.04 nm/RIU. (**b**) The amplitude variation of TFBG-SPR. The sensitivity is up to −1792.5 dB/RIU, obtained from the slope of the fitting curve.

To verify the viability of the biosensor, we tested this fiber SPR sensor using sodium chloride solutions with different refractive indexes (RIs). Sodium chloride solutions with different concentrations in deionized water were prepared as samples, and the RIs were measured with an Abbe refractometer (WAY-2S, accuracy of 0.001). In the experiment, sodium chloride solutions with RIs of 1.3241, 1.3388, 1.3459, and 1.3562 were pumped into the flow cell by a peristaltic pump (BT-100 2J, Longer) in turn. It is known that the large portion of the evanescent field of the hybrid plasmon wave propagating along the gold coating surface significantly enhances the sensitivity of the cladding modes located within the SPR position to the SRI. The SRI sensitivities of the TFBG wavelength and amplitude variations are shown in Figure 3a,b, respectively. The wavelength of the cladding mode obvious shifted toward the longer wavelength, while the RI increased from 1.3241 to 1.3562. It is shown that a good linear fitting curve is obtained for the SRI sensing range from 1.320 to 1.360 in Figure 3a. The result shows that the RI sensitivity of the SPR channel was 576.08 nm/RIU. Unfortunately, due to the limitation of the wavelength resolution of the OSA, it is hard to achieve an SRI resolution down to 10^−5^. Instead, we measured the amplitude change of the cladding mode of the TFBG-SPR sensor at the resonance wavelength. As shown in the inset of Figure 3b, the amplitude of the cladding mode near the SRP region reduced as the SRI increased with a high sensitivity. A small change of RI from 1.3310 to 1.3330 results in a notable and linear decrease in cladding mode amplitude from −16.329 to −19.914 dB. This shows that these amplitude changes can be used as the SPR response to RI, yielding a significant sensitivity up to −1792.5 dB/RIU.

**Figure 4:**
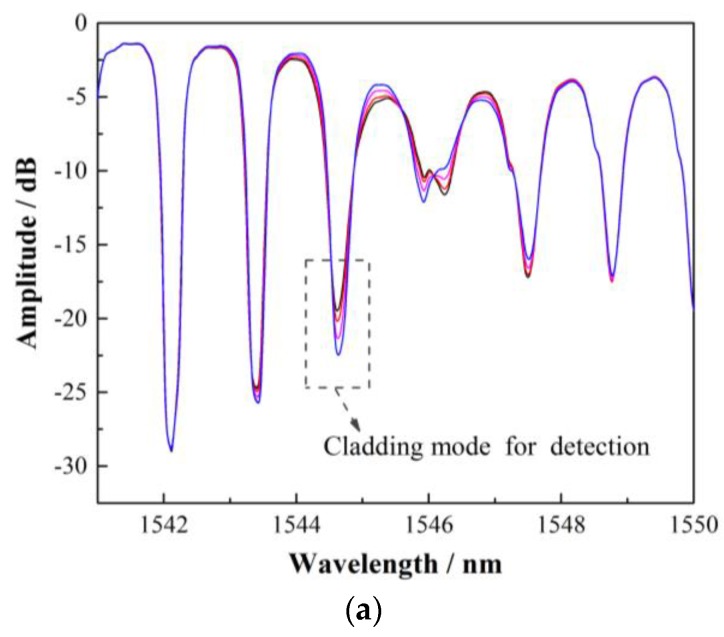

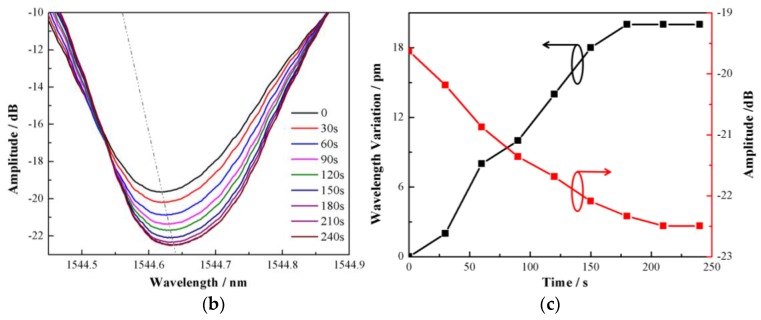
(**a**) The spectrum of the TFBG near the SPR resonance and the cladding modes selected for demodulation. (**b**) The spectral responses of the cladding mode next to the SPR resonances, and the reaction between boronic acid derivative and concanavalin A (Con A) was about 240 s. (**c**) The corresponding data analysis to monitor Con A via the developed TFBG-based sensor. The wavelength sensitivity was 20 pm/(mg/mL), while increasing the time of interaction results in a decrease of amplitude from −19.628 to −22.495 dB.

To demonstrate the feasibility of the TFBG-SPR sensor immobilized by boronic acid derivative for monitoring glycoprotein and compare the performance of the wavelength demodulation and amplitude demodulation, concanavalin A (Con A) was selected as a model protein. Con A contains a typical diols structure, which makes it suitable for boronic acid detection. Figure 4a,b present the spectrum of the TFBG near the SPR resonance and the spectral response of the selected cladding mode next to the SPR to monitor Con A. When the PBA binds the glycoprotein, the SPR resonance will shift according to the refractive index variation on the surface of the sensor resulting in the nearby cladding mode attenuation. It can be found that the wavelength sensitivity and amplitude sensitivity were 20 pm/(mg/mL) and 2.867 dB/(mg/mL), respectively. Considering the wavelength and amplitude resolution of the demodulation system (0.02 nm and 0.02 dB, respectively) and the stability of BBS (~0.01 dB), we used the amplitude demodulation method to detect the glycoprotein, which can improve the sensitivity. 

**Figure 5:**
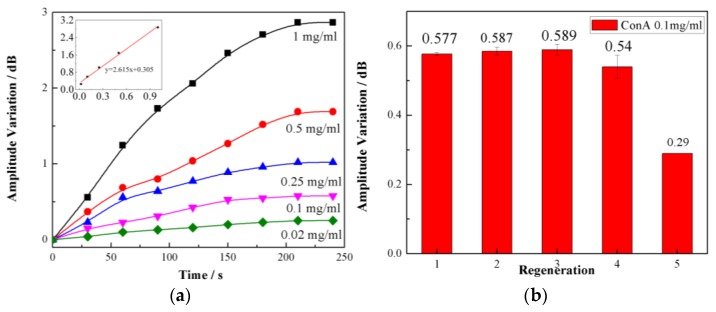
(**a**) The different concentrations of concanavalin A (Con A). The variation was 0.252 dB, 0.573 dB, 1.020 dB, 1.689 dB, and 2.867 dB when the concentration was 0.02 mg/mL, 0.1 mg/mL, 0.25 mg/mL, 0.5 mg/mL, and 1 mg/mL, respectively. (**b**) The regeneration of the developed TFBG-based sensor. The relative standard deviations were 0.69%, 2.44%, 2.79%, 5.76%, and 49.39%, respectively.

As shown in Figure 4, the dip amplitude of TFBG-SPR decreased due to the injection of Con A, which is consistent with the fact that Con A is bound easily and specifically to our designed PBA on the sensing surface. An ~3 dB variation in the amplitude was observed in the complex biochemical reaction process, which lasted 240 s. Based on the measurement method above, five different concentrations of Con A solutions (0.02, 0.1, 0.25, 0.5, and 1 mg/mL) were tested. As the concentrations increased, the amplitude decreased. As shown in the insets of Figure 5a, we also found that the amplitude variation showed a linear relationship with the concentrations of the sample. The calculated glycoprotein sensitivity of our sensor was 2.857 dB/(mg/mL) and the LOD was as high as 15.56 nM, which is calculated following the IUPAC criteria (3σ/M) and determined by the three times the standard deviation of the blank signal. This LOD is comparable to the last year’s reported results in [15,19,20,21], while our proposed TFBG-based fiber optical SRP sensing structure, to the best of our knowledge, is the first one to report a glycoprotein sensor combining a small functionalization molecular (instead of common proteins to catch glycoprotein) and optical fiber SPR platform working in mature communication band (compared with conventional SRP sensors working in visible band).. Our method possesses distinguished advantages of both SPR sensors and fiber sensors, including a simple and compact size, a low-cost system, label-free detection, and the ability to work in an optical communication band with a very cheap and mature demodulation method. Furthermore, the regeneration capability of our biosensor was demonstrated in Figure 5b. The boronic acid moiety in the ABA–PBA monolayer can covalently bond with 1,2- or 1,3-diols to form five- or six-membered cyclic complexes. By using a high-refractive-index solution (ethanol: PBS = 3:1, pH = 3), surface-bound Con A molecules are easily replaced by ethanol molecules in an acidic condition, effectively regenerating the sensing region, and the next concentration can be tested sequentially. In our experiment, when the concentration of Con A was 0.1 mg/mL, the sensor exhibited a good regeneration capability for up to 4 cycles. 

**Figure 6:**
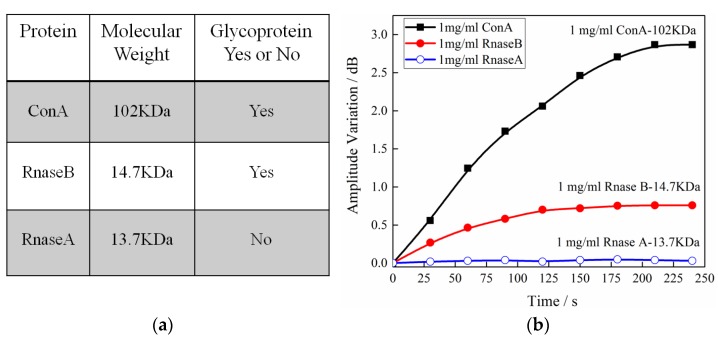
(**a**) Proteins that we have chosen in our experiment, Con A and Rnase B are glycoprotein, while the Rnase A and BSA are non-glycoprotein, all of these are to evaluate the selectivity of the biosensor. (**b**) The corresponding data analysis to monitor of different kinds of proteins via the developed TFBG-based sensor. The amplitude sensitivity was 2.867 dB/(mg/mL), 0.76 dB/(mg/mL), and 0.03 dB/(mg/mL).

To evaluate the binding selectivity of our sensor, four proteins (bovine serum albumin (BSA), Con A, Rnase B, and Rnase A) were chosen as the samples for testing. The molecular weight and types are given in the insets of Figure 6a. The bovine serum albumin (BSA) was used to block the unreacted carboxyl on the sensor surface, while Rnase A was employed to demonstrate the selectivity of the TFBG-SPR sensor. As for different kinds of glycoprotein, Con A and Rnase B demonstrate that the difference in molecular weight distinguishes the interaction of boronic acid derivative from that of different glycoproteins. As shown in the insets of Figure 6b, the boronic acid derivative effectively separated the glycoprotein from the non-glycoprotein under a consistent concentration. Additionally, the sensor has a negligible response to the non-glycoprotein. It also shows different responses depending on the molecular weight of the glycoprotein. As expected, boronic acid can more easily catch the “bigger” glycoprotein compared with the “smaller” one. 

## 4. Conclusions

In this paper, we proposed a new method to detect the glycoprotein by loading the synthesized long-chain boronic acid derivative on the surface of the SPR-TFBG sensors. After functionalizing the sensing region via chemical modification, the biosensor was used to detect the specific binding process between glycoprotein and boronic acid derivative. The sensor showed good selectivity between glycoproteins and non-glycoproteins as well as good regeneration ability. Using an amplitude demodulation method, a protein concentration sensitivity of 2.857 dB/(mg/mL) and an LOD of 15.56 nM were obtained. We also found that the sensor has a higher response to the glycoprotein with a higher molecular weight. The TFBG sensor operates in the common 1550 nm communication wavelength window so that the sensor demodulation can benefit from the mature and low-cost fiber-optic devices. Moreover, the sensor does not suffer from the temperature cross-sensitivity. Combining boronic acid and TFBG-SPR sensors provides a novel platform of detecting specific glycoproteins a compact size, a low cost, and a commercial, mature integration system that has good potential for applications in the study of cell-to-cell interactions and biological recognition. Future works will focus on improving the sensitivity and resolution by optimizing the sensor structure and functional materials.
